# Visual tracking in high-dimensional particle filter

**DOI:** 10.1371/journal.pone.0201872

**Published:** 2018-08-23

**Authors:** Jingjing Liu, Ying Chen, Lin Zhou, Li Zhao

**Affiliations:** Key Laboratory of Underwater Acoustic signal Processing of Ministry of Education, Southeast University, Nanjing, Jiangsu, China; Southwest University, CHINA

## Abstract

In this paper, we propose a novel object tracking algorithm by using high-dimensional particle filter and combined features. Firstly, the refined two-dimensional principal component analysis and the tendency are combined to represent an object. Secondly, we present a framework using high-order Monte Carlo Markov Chain which considers more information and performs more discriminative and efficient on moving objects than the traditional first-order particle filtering. Finally, an advanced sequential importance resampling is applied to estimate the posterior density and obtains the high-quality particles. To further gain the better samples, K-means clustering is used to select more typical particles, which reduces the computational cost. Both qualitative and quantitative evaluations on challenging image sequences demonstrate that the performance of our proposed algorithm is superior to the state-of-the-art methods.

## 1. Introduction

Visual object tracking is a fundamental research topic in computer vision, which plays a critical role in various applications, such as human computer interaction, driverless car, surveillance, and human motion analysis, to name a few. After several decades of visual tracking research, considerable progress has been made, it still remains very challenging for developing an all-situation-handled tracker that successfully handles all scenarios, such as partial occlusions, illumination changes, fast motions, camera motions, background clutter and viewpoint, etc.[1, 2]. Current visual tracking algorithms are classified as either generative or discriminative models. Both of them require filters to obtain the object’s candidates. Particle filter [3] also known as Sequential Monte Carlo filter which has been studied actively on object tracking, because it can cope with difficult nonlinear/non-Gaussian dynamic problems. Particle selecting is a matter of prime importance in the particle filter, which impacts the results of tracking objects immediately. Theoretically speaking, large number and high quality of particles could achieve the optimal approximation of the probability distribution [4]. However, the large number of particles means high computation costs such that the way to get high quality of particles is always complicated. Thus the tradeoff between the speed and the discriminative ability becomes a challenge problem to be solved.

The calculation of the particle tracking depends on the complexity of feature extraction and the amount of the sampling particles. Two kinds of methods are very often used to reduce the computation costs. One is to create the easier but more effective feature. For example, Perez introduced an ingredients incorporated multi-part color modeling and a background color model to handle color clutter in the background and complete occlusion of the tracked entities over a few frames[5]. Han presented an on-line appearance modeling technique based on sequential density approximation which adopted to variations of lighting condition, pose, scale and view-point over time [6]. Wang represented an object using 2DPCA which combined 2D basis matrices and an additional sparse error matrix [7]. Kong proposed a feature selection method which chose low dimension but more discriminative features [8]. Nevertheless, another alternative way is to reduce the quantity of samples. Ridge regression was employed [9] to decrease the computational costs for it could exclude the outlying particles. Their product sparse coding guaranteed their lower calculation simultaneously. Li reduced the number of particles considerably because of their effective sampling strategy [10]. Joint tracking algorithm adopted the mean shift and particle filter with model updating which could sample fewer particles and perform better performance for similar color appearance and cluttered backgrounds [11]. Shan improved the sampling efficiency using the mean shift optimization and their real-time hand tracking run fast as it used only color and motion cues [12].

Motivated by aforementioned discussions, this paper proposes a supplementary knowledge based high-order particle filtering tracking algorithm. The contributions of this work are threefold: (1) we represent the tracked object using the combined feature including an improved 2DPCA and the supplementary information. 2DPCA is a simple but effective feature which could achieve performance comparable to PCA with less computation costs. The supplementary information such as tendency could enrich the presentation of the object. (2) The high-order particle filter can be used to increase the algorithm’s accuracy because more information could be considered as well as more accurate and reliable moving object model could be supported. The traditional first-order Markov model is sensitive to loss of particle information from the previous time instant. For this reason, second-order object motion is widely used in tracking using Bayesian networks. However, it still cannot characterize the dynamics of moving objects. (3) Moreover, k-means clustering, a simple but valid algorithm, is adopted to selecting the sample particles with high possible appearance and further reduces the amount of the samples as it could decrease the computation costs.

The rest of the paper is organized as follows. In Section 2, we introduce our high-order particle filtering with combined features. Then, we present the summary of our proposed tracking algorithm in Section 3. Section 4 explains experimental results and analysis on tracking. Finally, Section 5 gives the conclusions.

## 2. High-order particle filtering with combined features

We begin with a concise review of particle filtering and then introduce our high-order particle filter tracking framework.

### 2.1 Review of particle filtering

**Fig 1:**
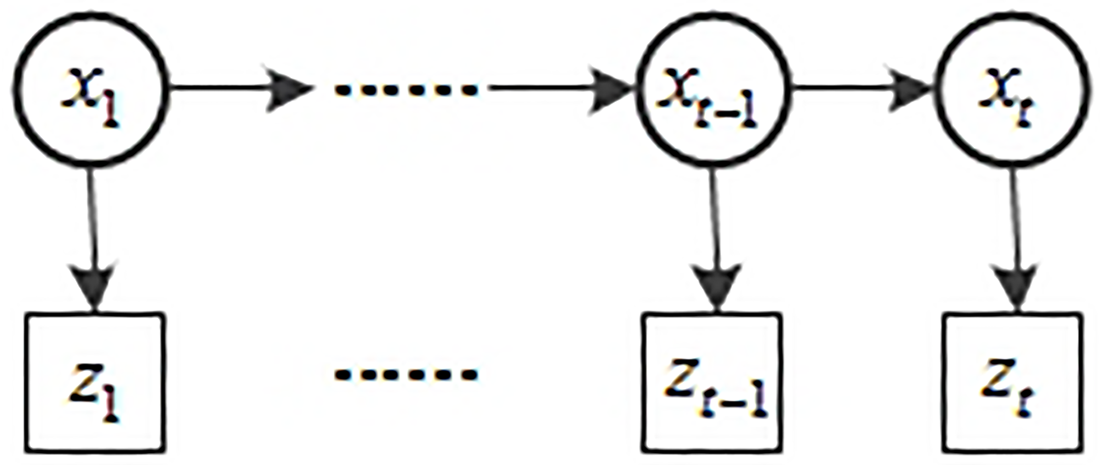
Dynamic Bayesian network structure of particle filtering.

Particle filter is a filtering method which has been shown to offer improvements in performance over non-linear or non-Gaussian environment. The traditional particle filter is derived on the first-order Markov chain. Its basic network structure is shown in Fig 1.

Fig 1 represents the structure of the particle filters (PF). The state parameter vector of a target and its observation at time t is denoted as *x*_*t*_ and *z*_*t*_, respectively. The history of observations from time 1 to *t* is denoted as *Z*_*t*_ = {*z*_1_, ⋯, *z*_*t*_}. The state-space model is a first-order Markov chain and the current state *x*_*t*_ only depends on the previous state *x*_*t*−1_. However, particle filters using the first-order Markov model cannot perform on fast moving objects efficiently. The situation would be worse if the object from the previous time instant is lost. Therefore, the high-order Markov Chain named as m-th-order Markov Chain is required to model the moving objects with high-order dynamics. Compared with the state-space model of the first-order particle filters, an m-th-order Markov chain’s current state *x*_*t*_ depends on the past *m* states and we have

$$p({\text{x}}_{t}|{\text{x}}_{t-1,}{\text{x}}_{t-2,}\ldots ,{\text{x}}_{0})=p({\text{x}}_{t}|{\text{x}}_{t-1,}{\text{x}}_{t-2,}\ldots ,{\text{x}}_{t-m}).$$ (1)

### 2.2 Probability propagation of high-order particle filtering

In particle filtering, the tracking only uses sequential state probability propagation information. The information derived from the states and the objects’ prior is also quite useful to influence the sampling strategies and the tracking. In this work, we assume that a target’s appearance is modeled by a subspace model and the state space is augmented with the corresponding appearance’s supplementary information such that the state *x*_*t*_ = (*c*_*t*_, *s*_*t*_) consists of two parts: the state *c*_*t*_, which models the estimated position, and the supplementary information *s*_*t*_, containing the moving tendency and so on. The posterior distribution now is described as *P*(*c*_*t*_, *s*_*t*_|*Z*_*t*_). Unlike the probability *P*(*x*_*t*_│*Z*_*t*_, *A*) in the Appearance-Guided Particle Filtering [13], in which the tracking employs further prior of object appearance, we show the solutions using the assist knowledge derived between the sequential states.

**Fig 2:**
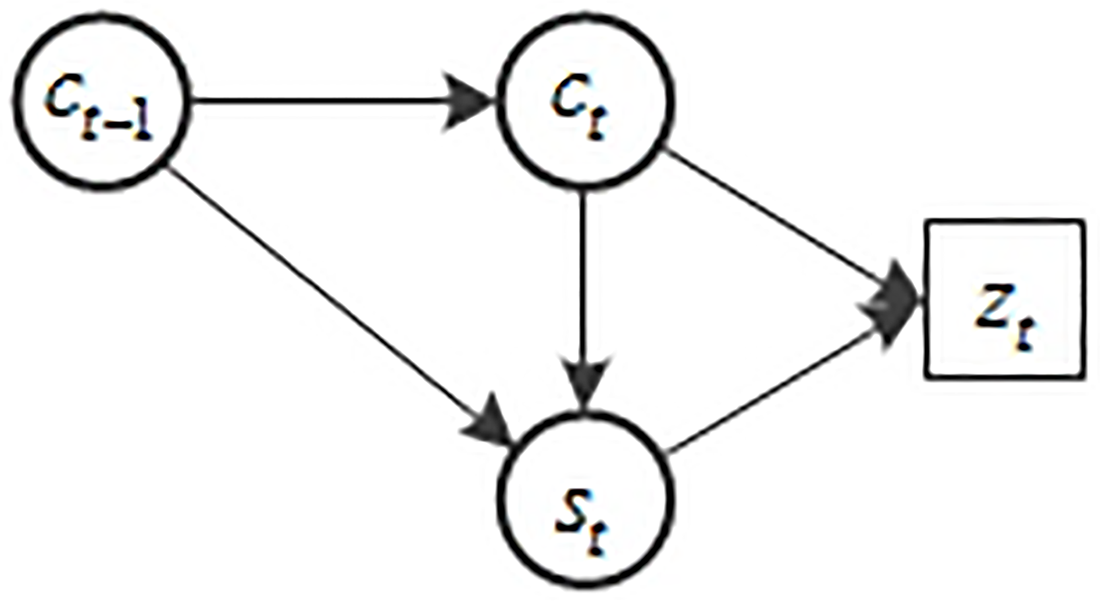
Bayesian network structure of our first-order framework.

Fig 2 shows our first-order framework’s Bayesian network structure and the network becomes:

$$P({\text{c}}_{t},{\text{s}}_{t}|{\text{Z}}_{t})=kP(Z_{t}|{\text{c}}_{t},{\text{s}}_{t})\times \int {}_{c_{t-1}}\int {}_{s_{t-1}}P(c_{t},{\text{s}}_{t}|{\text{c}}_{t-1},{\text{s}}_{t-1})P({\text{c}}_{t-1},{\text{s}}_{t-1}|Z_{t-1})$$ (2)

The posterior over the current state is influenced by integrating the target state and the assist knowledge at the previous time-step. Once we integrate out the assist part and approximate the filter using a hybrid Monte Carlo Method, the Bayes filter is reduced to Rao-Blackwellized particle filter (RBPF) [14] and the filter would be:

$$P({\text{c}}_{t}|{\text{Z}}_{t})=k\int_{{\text{s}}_{t}}P(Z_{t}|c_{t},{\text{s}}_{t})\times \int {}_{c_{t-1}}\int {}_{s_{t-1}}P(c_{t},{\text{s}}_{t}|{\text{c}}_{t-1},{\text{s}}_{t-1})P(c_{t-1},{\text{s}}_{t-1}|Z_{t-1}).$$ (3)

In a Rao-Blackwellized particle filter, the assumption that the moving model for the state does not depend on the assist information is necessary and the marginal Bayes filter is obtained as follows:

$$P({\text{c}}_{t}|{\text{Z}}_{t})\approx \text{k}\sum_{i}w_{t-1}^{\left(i\right)}P({\text{c}}_{t}|{\text{c}}_{t-1}^{\left(i\right)})\times \int {}_{s_{t}}P({\text{Z}}_{t}|c_{t},{\text{s}}_{t})\int {}_{s_{t-1}}P({\text{s}}_{t}|c_{t},{\text{c}}_{t-1}^{\left(i\right)},{\text{s}}_{t-1}){\text{s}}_{t-1}^{\left(i\right)}({\text{s}}_{t-1}).$$ (4)

**Fig 3:**
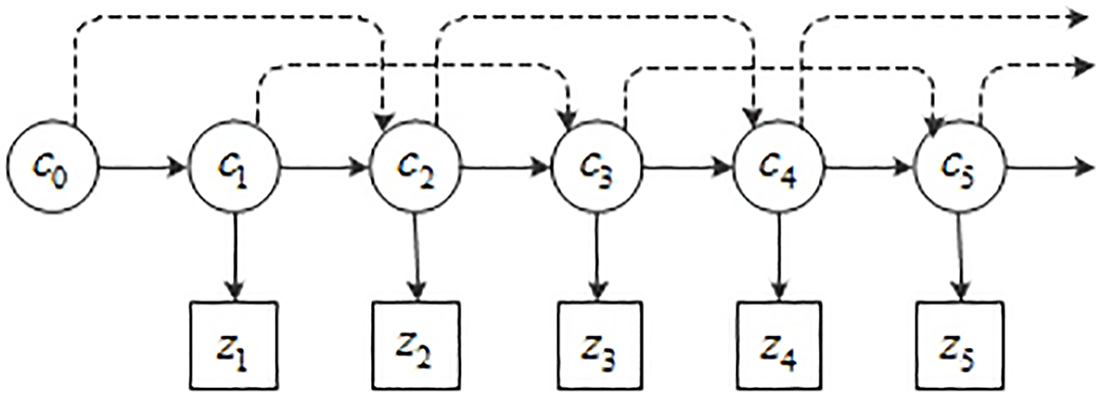
Second-order hidden Markov model.

The approximation to this Bayes network model need an assumption that the motion model for the location at time *t* does not depend on the previous time-step’s knowledge but uses the same importance sampling schemes as with the particle filter. However, if assuming the assist knowledge is independent of the state at the previous time-step and only influence the Bayesian bootstrap or the results of sampling importance resampling, the aforementioned model would degenerates to a BN structure for particle filtering. Hence, we investigate the probability *P*(x_*t*_ | Z_*t*_) as follows:

$$P(x_{t}|{\text{Z}}_{t})\approx P({\text{c}}_{t}|{\text{Z}}_{t})\propto P({\text{Z}}_{t}|{\text{c}}_{t})\int P({\text{c}}_{t}|c_{t-1})\cdot P(c_{t-1}|{\text{Z}}_{t-1}){\text{dc}}_{t-1}.$$ (5)

where a first-order Markov chain of the states is considered. In order to better track the fast moving objects, high-order Markov Model is adopted and its posterior density function *P*(*C*_*t*_ | *Z*_*t*_) is shown in formula 6. Fig 3 provides an example of a second-order hidden Markov model which could give more intuitionistic descriptions. The circle nodes and the square nodes denote the states of the object and the observations, respectively.

$$\begin{array}{l} P({\text{C}}_{\text{t}}|{\text{Z}}_{t})=\frac{p({\text{z}}_{t},{\text{C}}_{t},{\text{Z}}_{t-1})}{p({\text{z}}_{t},{\text{Z}}_{t-1})}\\ =\frac{P({\text{z}}_{t}|{\text{C}}_{t},{\text{Z}}_{t-1})P({\text{C}}_{t},{\text{Z}}_{t-1})}{P({\text{z}}_{t},{\text{Z}}_{t-1})}\\ =\frac{P({\text{z}}_{t}|{\text{C}}_{t},{\text{Z}}_{t-1})P({\text{C}}_{t}|{\text{Z}}_{t-1})}{P({\text{z}}_{t}|{\text{Z}}_{t-1})}\\ =\frac{P({\text{z}}_{t}|{\text{C}}_{t},{\text{Z}}_{t-1})P({\text{c}}_{t},{\text{C}}_{t-1}|{\text{Z}}_{t-1})}{P({\text{z}}_{t}|{\text{Z}}_{t-1})}\\ =\frac{P({\text{z}}_{t}|{\text{C}}_{t},{\text{Z}}_{t-1})P({\text{c}}_{t}|{\text{C}}_{t-1},{\text{Z}}_{t-1})P({\text{C}}_{t-1}|{\text{Z}}_{t-1})}{P({\text{z}}_{t}|{\text{Z}}_{t-1})}\\ \propto P({\text{z}}_{t}|{\text{C}}_{t},{\text{Z}}_{t-1})P({\text{c}}_{t}|{\text{C}}_{t-1},{\text{Z}}_{t-1})P({\text{C}}_{t-1}|{\text{Z}}_{t-1})\\ =P({\text{z}}_{t}|c_{t})P({\text{c}}_{t}|c_{t-m:t-1})P({\text{C}}_{t-1}|{\text{Z}}_{t-1}) \end{array}.$$ (6)

### 2.3 Weight updating of the high-order particle filtering

As aforementioned, the basic Sequential Importance Resampling (SIR) algorithm given starts from a random measure with equal weight on each of the *N* sample values and samples *N* times independently from the set with probabilities to obtain an equally weighted random measure. Exploiting the SIR method, the high-order particle filtering’s posterior density can be estimated as

$$P({\text{C}}_{t}|{\text{Z}}_{t})\approx \sum_{i=1}^{N}\omega_{t}^{i}\delta ({\text{C}}_{t}-{\text{C}}_{t}^{i})$$ (7)

The weight update equation is given by the equation

$$\omega_{t}^{i}\propto \frac{P({\text{z}}_{t}|{\text{c}}_{t}^{i})\text{P}({\text{c}}_{t}^{i}|{\text{c}}_{t-m:t-1}^{i})\text{P}({\text{c}}_{0:t-1}^{i}|{\text{Z}}_{t-1})}{Q({\text{c}}_{t}^{i}|{\text{c}}_{t-m:t-1}^{i},{\text{z}}_{t})Q({\text{c}}_{0:t-1}^{i}|{\text{Z}}_{t-1})}=\omega_{t-1}^{i}\frac{P({\text{z}}_{t}|{\text{c}}_{t}^{i})\text{P}({\text{c}}_{t}^{i}|{\text{c}}_{t-m:t-1}^{i})}{Q({\text{c}}_{t}^{i}|{\text{c}}_{t-m:t-1}^{i},{\text{z}}_{t})}$$ (8)

where $P(z_{t}|c_{t}^{i})$, $P(c_{t}^{i}|c_{t-m:t-1}^{i})$ and $Q(c_{t}^{i}|c_{t-m:t-1}^{i},z_{t})$ are the likelihood, the transition probability and the importance density, respectively. Hence, the posterior filtered density $P(c_{t-m+1:t}^{i}|Z_{t})$ can be shown as

$$P({\text{c}}_{t-m+1:t}|{\text{Z}}_{t})\approx \sum_{i=1}^{N}\omega_{t}^{i}\delta ({\text{c}}_{t-m+1:t}-{\text{c}}_{t-m+1:t}^{i})$$ (9)

For more details of the derivation, we refer readers to [1]. However, this SIR filter is vulnerable to sample impoverishment, so that the particle distribution cannot give an accurate approximation of the required PDF. Usually, researchers explore large numbers of particles in realistic applications which require more computation. For the sake of reducing computational complexity of particle filters, unequal importance weights measure before resampling is employed to refine the SIR strategy. Then, the importance density $Q(c_{t}^{i}|c_{t-m:t-1}^{i},z_{t})$ would be changed to $\omega_{t-1}^{i}Q(c_{t}^{i}|c_{t-m:t-1}^{i},z_{t})$ as the new one. The obvious distinction between the refined density $\omega_{t-1}^{i}Q(c_{t}^{i}|c_{t-m:t-1}^{i},z_{t})$ and the original density $Q(c_{t}^{i}|c_{t-m:t-1}^{i},z_{t})$ is that the refined density adds $\omega_{t-1}^{i}$. The weighted sample points carry more information than an equal number of unweight points. We apply $\omega_{t-1}^{i}$ to our high-order particle filter because its superiority to the SIR filter both in terms of combating sample impoverishment and in computational cost. The paper [15] is recommended to the readers for more comprehensive and profound understanding to the weighted sample points. Combing the preceding ideas, the proposed high-order particle filter algorithm is presented in Algorithm 1.

**Algorithm 1. Proposed Advanced High-order Particle Filtering Algorithm**

Initialize: Start from a random measure with M support points ${\left\{c_{0}^{i},\omega_{0}^{i}\right\}}_{i=1}^{M}$, obtained by stratified sampling, which approximates to the PDF *P*(c_0_)

For *t* = 1, 2, 3…

 For *i* = 1, 2, …, *M*

  Keep particles $c_{t-m:t-1}^{i}$

  Draw particles $c_{t}^{i}∼\omega_{t-1}^{i}Q({\text{c}}_{t}^{i}|{\text{c}}_{t-m:t-1}^{i},{\text{z}}_{t})$

  Calculate the importance weight $\omega_{t}^{i}$ as in formula (8)

 End for *i*

 Normalize the importance weight $\omega_{t}^{i}$ according to $\omega_{t}^{i}/\sum_{i=1}^{M}\omega_{t}^{i}$

 Estimate *c*_*t*_ according to $\sum_{i=1}^{M}\omega_{t}^{i}c_{t}^{i}$

 Resample ${\left\{{\text{c}}_{t-m+1:t}^{i},\omega_{t}^{i}\right\}}_{i=1}^{M}$

End for *t*

### 2.4 Transition probability $P(c_{t}^{i}|c_{t-m:t-1}^{i})$ and importance density $Q(c_{t}^{i}|c_{t-m:t-1}^{i},z_{t})$

Compared with the dynamics of the objects from the traditional first-order particle filter, that is given as *x*_*t*_ = *ax*_*t*−1_ + *bv*_*t*_, our dynamics of the tracking object is assumed as *c*_*t*_ = *Ac*_*t*−*m*_ + ⋯ + *Ec*_*t*−2_ + *Fc*_*t*−1_ + *Gv*_*t*_, where *v*_*k*_ is modeled as Gaussian distribution *μ*(0, *Σ*_1_) and *Σ*_*1*_ is diagonal covariance metrics. In order to calculate the coefficients, maximum-likelihood estimation method is used. The transition probability is given as follows:

$$P({\text{c}}_{t}^{i}|{\text{c}}_{t-m:t-1}^{i})=N_{c_{t}^{i}}(Ac_{t-m}^{i}+\cdots +Ec_{t-2}^{i}+Fc_{t-1}^{i},G^{2}\sum_{1})$$ (10)

where *N*_*c*_(*μ*, Σ) = (1) / (2*π*|Σ|)exp(−(1) / (2)(c − *μ*)^*T*^ Σ^−1^(c − *μ*)). After obtaining the coefficients, the new samples based on the previous samples could be generated intuitively and the notation is as follows:

$$c_{t}^{i}=Ac_{t-m}^{i}+\cdots +Ec_{t-2}^{i}+Fc_{t-1}^{i}+Hv_{t}$$ (11)

Note that the value of *H* is the variance of the importance density function. Similar to the transition probability, the importance density could be given as:

$$Q({\text{c}}_{t}^{i}|{\text{c}}_{t-m:t-1}^{i},{\text{z}}_{t})=N_{c_{t}^{i}}(Ac_{t-m}^{i}+\cdots +Ec_{t-2}^{i}+Fc_{t-1}^{i},H^{2}\sum_{1})$$ (12)

### 2.5 “Extra Step”: Analytical update

We have laid the theoretical foundation for the high-order particle filter and derived the probability propagation that is related to the BN as shown in Fig 3. However, our supplementary information is not covered and the valuable information can afford more prove to generate more precise particles. How to apply the supplementary information better is a confusing problem for us. It is widely accepted that large numbers of particles which distribute reasonably could improve the performance of the trackers. Chang explored a mixture distribution which generated two sets of samples from *P*(*x*_*t*_|*x*_*t*−1_) and *P*(*x*_*t*_|*A*), respectively [14]. This method combines particles with both dynamic model-driven information and appearance information and it is effective for visual tracking problems such as articulated hand tracking and lip-contour tracking. The amount of its particles in combined methods is more than the one in dynamic model which implies high computational cost. However, our original intention is to reduce the computation cost of implementing particle filters which means less particles. Huang used ridge regression to delete the outlying particles to obtain fewer particles [9]. Combining the two different methods above, here, we use *P*(*c*_*t*_, *s*_*t*_|*Z*_*t*_) to reselect more suitable particles. As shown aforementioned, in order to simplify the model, *M* particles have been generated using the advanced SIR algorithm and only cover the information of the state $c_{k-m+1:k}^{i}$. Moreover, the supplementary information *s*_*t*_ is an important resource to resample the particles. The mixture distribution of *P*(*c*_*t*_, *s*_*t*_|*Z*_*t*_) is set according to the following equation:

$$P({\text{c}}_{t},{\text{s}}_{t}|{\text{Z}}_{t})=\alpha P({\text{c}}_{t}|{\text{Z}}_{t})+(1-\alpha )P({\text{s}}_{t}|{\text{Z}}_{t})$$ (13)

Note that the probability *P*(*c*_*t*_|*Z*_*t*_) and *P*(*s*_*t*_|*Z*_*t*_) is caused by the state transition and the supplementary information transition, respectively. When *α* is set to one, it degenerates to the original particle filtering in which only the dynamical information is used. Form Algorithm 1, we have set *M* particles using the state transition. Similar to generate *M* particles, *M*_*s*_ particles would be generated based on *P*(*s*_*t*_|*Z*_*t*_). Here we use the direction of motion as the object’s the supplementary information between the frames. Then, the two sets of particles are combined together as a complete sample set whose amount is more than each of them. Since the number of the particles is large, it must exists a method to reduce the computational cost for the sake of obtaining our expected idea. K-means clustering is applied in our method to reduce the amount of sample particles, which is simple but useful to decrease the computational cost. K-means clustering is popular for cluster analysis and aims to partition *N* observations into *k* clusters in which each observation belongs to the cluster with the nearest mean. Expressing with the mathematical language, the objective of K-means clustering is to find:

$$\underset{S}{\text{arg}\;\text{min}}\sum_{i=1}^{k}\sum_{x\in S_{i}}{\left\|x-\mu_{i}\right\|}^{2}$$

Note that each of observations in the observation sets (*x*_1_, *x*_2_,⋯ *x*_*n*_) is a *d*-dimensional real vector and would be partitioned into *k*(≤ *n*) sets *S* = (*S*_1_, *S*_2_, ⋯*S*_*n*_), and *μ*_*i*_ is the mean of points in *S*_*i*_. One of the most popular heuristics for solving the k-means problem is generalized Lloyd’s algorithm. In order to implement Lloyd’s algorithm simply and efficiently, the filtering algorithm which is based on storing the multidimensional data points in a kd-tree is applied. A kd-tree represents a hierarchical subdivision of the point set using axis aligned splitting hyperplanes. Given *n* observations, this produces a tree with *O(n)* nodes and *O*(*log n*) depth. After using filtering algorithm, the observation set is partitioned into k clusters with its corresponding cluster center. For each cluster, we select half of the observations which are near to the center of each set. Except the sample method using the special distance from the center, a randomly sample method is another useful and practicable way. Certainly, mixed selecting method combining the specific scheme with the random way is definitely doable. Here, the points near the center are selected for the following actions because we do not cut the number of the sample particles so aggressively. Therefore, the amount of the particles would be reduced to half of the original one.

## 3. Proposed tracking algorithm summary

This tracking algorithm is under the frame of high order particle filter which can be viewed as a Bayesian inference task in a Markov model with hidden state variables. In this paper, the feature extraction is not the most important part in the whole tracking system. Principal component analysis (PCA) is a classical dimension reduction method, which has been used as a method to extract feature in many areas. The advanced PCA which is called two-dimensional principal component analysis (2DPCA) attracts more attention because of its better performance and less computational cost. Motivated by the advantage of 2DPCA, we represent an object by using 2D matrices. For a series of image matrices *z* = (*z*_1_, *z*_2_, ⋯ *z*_*k*_), an orthogonal left-projection matrix $U\in R^{d_{l}\times k_{l}}$, an orthogonal right-projection matrix $V\in R^{d_{r}\times k_{r}}$, and a projection coefficients *Co* = (*Co*_1_, *Co*_2_, ⋯ *Co*_*k*_) is obtained by solving the objective function ${\text{min}}_{U,V,{\text{Co}}_{i}}\frac{1}{k}\sum_{i=1}^{k}{∥z_{i}-U{Co}_{i}V^{T}∥}_{F}^{2}$. The optimizations of the orthogonal left-projection matrix *U* and the orthogonal right-projections matrix *V* are computed according to the algorithms in the paper [16] and [17] respectively. Then *Co*_*i*_ could be approximated using *U*^*T*^ *z*_*i*_*V*. After the projection coefficient is calculated, the likelihood can be obtained by the reconstruction error,

$$P({\text{z}}^{i}|{\text{c}}^{i})=\text{exp}(-\|z^{i}-{UCo^{i}V^{T}\|}_{F}^{2})$$ (14)

We summarize the algorithm in Algorithm 2.

**Algorithm 2. Proposed Tracking Algorithm**

Initialize: an observation Matrix *Z*, left- and right- projection matrices *U* and *V*.

Given a random measure with *M* support points ${\left\{{\text{c}}_{0}^{i},\omega_{0}^{i}\right\}}_{i=1}^{M}$, the following steps are performed to construct a new set of samples.

1. select *M* particles using the proposed advanced high-order particle filtering algorithm

2. select *N* particles using the supplementary information

3. the distribution of *M+N* particles is according to *P*(c_*t*_, s_*t*_|Z_*t*_)

4. k-means cluster is used to select the more suitable particles

5. measure each particles using $P({\text{z}}^{i}|{\text{c}}^{i})=\text{exp}(-\|z^{i}-{UCo^{i}V^{T}\|}_{F}^{2})$

6. choose the best candidate as the current state

## 4. Experiments and analysis

The proposed algorithm is implemented in Matlab (R2013a) on a personal computer Inter(R) Core(TM) i5-4300U 1.90GHz CPU with 4 GB RAM. The object is initialized manually and the proposed method can process at about 10 frame/s. In order to evaluate the performance of the proposed tracking framework, 13 image sequences are tested in the experiments. These sequences are public which can be obtained from the internet easily and the website address is http://www.visual-tracking.net. Such videos cover most challenging conditions in visual tracking, scale variation, occlusion, rotation, motion blur, background clutter, illumination variation and fast motion.

### 4.1 Ablation study

In order to demonstrate the feasibility of the proposed high-dimensional methods, we summarize the performance of our proposed tracker and the trackers without some variants in Table 1. 2DPCA tracking is a base tracker and its MATLAB source codes could be downloaded on http://ice.dlut.edu.cn/lu/publications.html. The tracker with combined features here is an alternation of the 2DPCA tracker which changes the 2DPCA feature of the original tracker into the combined features and the combined features comprise 2DPCA and tendency. The third tracker named 2DPCA_HDPF is built by changing the *l*-1 regulation of the 2DPCA tracker into high-order particle filter. The combined features_HDPF tracker is made by abandoning the k-means cluster from the proposed tracker and it is also can be written as ours_without cluster tracker. Throughout the comparisons between the 5 trackers, it is easily concluded that the proposed tracker performs the best and all the parts of the proposed tracker are useful to improve the performance. Noticed that ours performs better than ours_without cluster and it only rises about 0.4 because the difference between the two trackers is a k-means cluster method. The proposed algorithm runs faster than the combined features_HDPF tracker.

**Table 1:** Comparison in terms of success rate.

Tracker	2DPCA	Combined features	2DPCA_HDPF	Combined features_HDPF	Ours
Average	81.73	83.92	85.64	89.8	90.19

**Fig 4:**
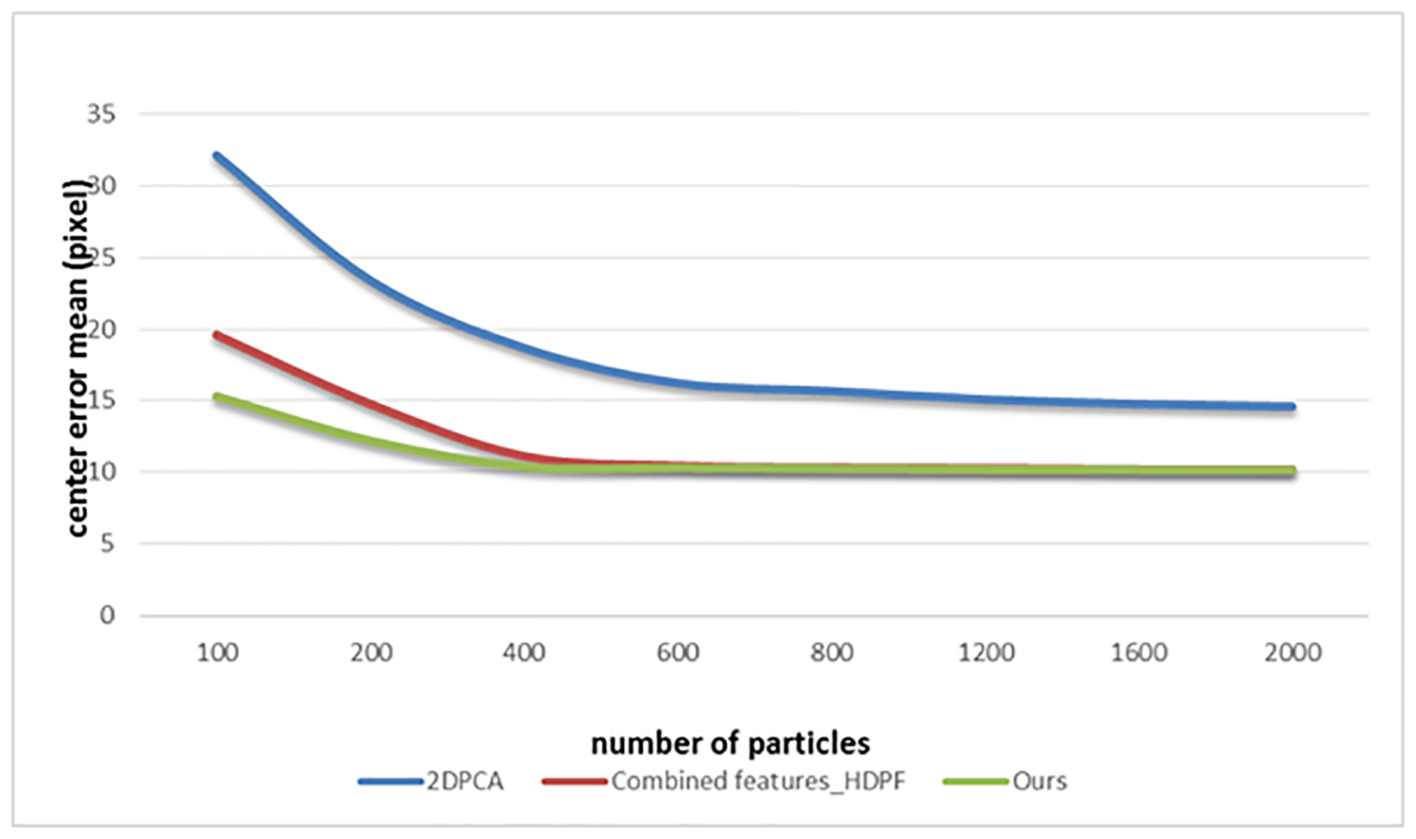
Center error mean with different sampling particles.

To further illustrate the efficiency of the proposed method, the experiments on tracking accuracy versus number of particles have been made subsequently. Three different trackers are selected to participate the experiments and those are the 2DPCA tracker, the Combined features_HDPF tracker and ours. Fig 4 shows the average center error mean with different number of particle sampling for each tracker and its general trend appears to be decrease with increasing number of sample particle. However, the average center error mean cannot drop steadily but remains almost constant when the number of sample particle is more than 800. The proposed method could achieve an acceptable performance when the number of sample particle is 400. In the paper, we set 400 as the number of the particle sampling which reaches the balance between the computation and the performance.

### 4.2 Comparison with state-of-the-art trackers

We evaluate our tracker against 13 state-of-the-art algorithms, including IVT tracking [18], MIL tracking [19], DFT tracking [20], L1APG tracking [21], ASLA tracking [22], DLT tracking [23], SCM tracking [24], 2DPCA tracking [15], SCT tracking [8], TLD tracking [25], VTD tracking [26], Struck tracking [27] and SPC tracking [28]. The source codes for all the evaluated trackers can be downloaded from the internet. For fair comparisons, all the evaluated trackers are initialized with the same parameters.

For the purpose of assessing the performance of the proposed tracker, we conduct quantitative comparisons between the proposed method and other algorithms using the PASCAL VOC criterion score [29]. Table 2 shows that the proposed method can achieve an excellent tracking result in most sequences in terms of both the average and the standard of center error. Even so, the proposed algorithm improves the performance about 5.8 and 6.7 pixels compared with the tracker which is second best. In addition, Table 3 shows the success rate provided by our proposed tracker and other approaches on 13 sequences. The proposed tracker shows the optimal or suboptimal performance in almost all the sequences, which obtains a mean success rate of 90.19%. However, our method runs slower than SCT. Although ours is either the most accurate or the fastest one, ours do the best within an acceptable scale.

**Table 2:** Comparison in terms of the center error mean and standard deviation (in pixel).

	IVT	MIL	DFT	LIAPG	SCM	ASLA	DLT	SCT	2DPCA	TLD	VTD	Struck	SPC	OURS
**Car4**	2	50.8	92.5	77	4.1	1.7	2.3	3.1	4.3	11.3	56.4	68	80	**1.4**
	1.6	37.2	59.6	52.8	4.3	1	1.2	1.8	3.7	8.6	49.2	56.3	67.1	**0.8**
**CarDark**	8.2	43.5	17.4	1.4	**0.9**	1	80.7	2.9	3.5	21.2	16.2	1	1.2	1
	11.1	37.2	22.7	1	0.9	0.9	52.3	2.4	2.7	19.6	15.3	**0.8**	1	0.9
**Cliffbar**	74.3	57.2	46.8	54.4	49	29.8	**2.2**	22.3	23.8	30.4	36.4	3.7	11.6	6.1
	33.6	34.1	34.9	46.1	39.8	28.7	**2.8**	30	25.7	31.7	32.1	2.9	9.8	9.1
**Cup**	63.1	4.6	3.2	3.2	**2.2**	**2.1**	**2.1**	2.5	2.5	4.1	2.7	8.3	4.9	2.5
	40.5	3.7	1.7	3.7	1.1	**1**	**1**	1.3	1.2	4.9	1.6	6.9	4.2	**1**
**FaceOcc1**	7.4	30.9	4.3	7	**3.7**	83.8	6.5	4.8	5.9	6.3	3.9	4.8	4.1	**3.7**
	6.2	19.8	3.8	4.7	2.3	51.1	3.1	2.8	3	5.6	2.4	3.9	3.2	2.1
**FaceOcc2**	7.7	14.5	7.4	13.3	7.6	17.4	7.8	6.4	10.2	32.1	12.7	8.9	30.4	**5.9**
	5.3	8.2	3.7	13.5	7.1	30.1	6.4	5.4	7	26.4	9.1	6.7	26	**3**
**Football**	14.6	12.1	30	15	16.5	15.2	15.6	13.5	7.3	36.3	**6.3**	45.8	16.2	7.6
	18.4	15.5	50.1	19.8	17.7	18.7	15.5	17.1	**4.7**	45.8	5.1	52.1	15	5.4
**Jogging1**	86	96.3	**51.1**	89.9	132.4	104.1	111.7	113.7	78	71	65	107.4	129	71.7
	49.1	56.6	**25.5**	53.6	81.5	60.7	65.6	68	62.7	64.3	61.4	89	100.4	60.1
**Jumping**	62.2	6.3	58.3	87.6	69.1	50.4	104.8	**5.5**	6.1	6.7	53.7	32.1	19	5.9
	34.7	3.2	26.6	60	49.3	45.5	44.9	1.7	2	4	38.2	38.4	24	**1.6**
**Man**	42.2	56.5	37.9	26.1	**1.1**	1.3	1.2	1.6	1.5	2.7	2.3	**1.1**	1.3	**1.1**
	22.8	15.5	20.6	15.6	0.6	0.6	**0.5**	0.7	0.6	1.4	1.7	**0.5**	0.6	**0.5**
**Subway**	135.6	135.2	3.4	146.5	3.9	**3.3**	166.1	124	45	41.4	80	32.9	62	5.6
	94.8	91.7	**1.7**	107.3	2.1	2.5	84.5	85.7	39.2	39.2	69.2	26.1	71	4.3
**Suv**	57.7	82.2	111.4	93.1	4.2	74.7	20	5.1	6.1	67.3	42.3	72.1	89	**3.8**
	55.1	60.2	57.9	105.4	5.3	93.4	40.6	6.3	9.5	7	37	66	76.3	**4.6**
**Trellis**	119.6	71.5	45.5	62.3	7.3	7.8	25.6	4	17	41.2	66.2	54.2	72	**3.6**
	98.3	53.8	46.7	44.3	10.7	15.2	17.2	3.3	21	37	59	60	66.7	**2.8**
**Average**	52.35	50.89	39.18	52.06	23.22	30.2	42.04	20.33	16.25	28.62	35.75	33.87	40.05	**10.38**
	36.27	33.59	27.34	40.6	17.13	26.87	25.81	17.42	14.08	22.73	28.64	31.51	35.8	**7.39**

**Table 3:** Comparison in terms of success rate.

	IVT	MIL	DFT	L1APG	SCM	ASLA	DLT	SCT	2DPCA	TLD	VTD	Struck	SPC	OURS
**Car4**	**100**	27.92	24.73	29.89	97.42	**100**	95.9	**100**	91	71	37	32	24	**100**
**CarDark**	72.26	18.58	68.96	**100**	99.75	**100**	12.72	95.67	92.3	63	73	**100**	**100**	97.2
**Cliffbar**	1.91	0.21	24.36	36.44	18.01	45.34	**72.42**	59.96	51.6	45.78	31.2	19.57	52.3	65.1
**Cup**	24.42	94.72	**100**	88.12	**100**	**100**	**100**	**100**	**100**	87	96	74	82	**100**
**FaceOcc1**	99.78	60.54	**100**	**100**	**100**	25.67	**100**	**100**	**100**	94	**100**	97	**100**	**100**
**FaceOcc2**	98.15	78.33	99.63	82.14	92.86	80.67	78.69	93.1	87.4	61	87	94	65	**100**
**Football**	69.34	74.31	75.69	71.27	58.56	65.47	56.35	77.9	**87.6**	27	76.1	14.83	58	81.3
**Jogging1**	22.48	22.48	21.5	22.15	21.17	22.48	22.48	21.5	23.2	32.4	**48.3**	27.9	16.9	45.7
**Jumping**	12.14	96.17	15.02	12.14	12.14	17.89	12.78	**99.36**	98.6	46.9	62.1	72.8	83.65	99.13
**Man**	23.88	0.75	21.64	25.37	**100**	**100**	**100**	**100**	**100**	92	95	**100**	**100**	**100**
**Subway**	20.57	2.86	**99.43**	22.29	**99.43**	98.86	5.71	21.71	63	73	54.6	83.2	66.2	99.21
**Suv**	44.34	13.12	5.19	52.7	98.52	58.2	81.06	96.93	96.8	41.2	73.6	37.1	25.76	**98.87**
**Trellis**	32.69	12.3	51.67	4.75	85.24	85.41	27.42	72.06	71	57.8	31.3	41.2	36.2	**86**
**Average**	47.84	37.64	54.45	49.79	75.62	69.23	58.89	79.86	81.73	60.93	66.55	61.05	62.31	90.19

## 5. Conclusions

In this paper we propose a high degree particle filter with the methods to reduce the particles for robust visual tracking. We represent the tracked object by 2DPCA and the tendency of the object and model tracking under the frame of high degree particle filter. In order to reduce the computational cost, K-means cluster is used to select the more suitable particles. Then, reconstruction error is adopted to judge the best candidate. Experiments on challenging video clips demonstrate the robustness of the proposed algorithm.
